# A Tunable-Gain Transimpedance Amplifier for CMOS-MEMS Resonators Characterization

**DOI:** 10.3390/mi12010082

**Published:** 2021-01-15

**Authors:** Rafel Perelló-Roig, Jaume Verd, Sebastià Bota, Jaume Segura

**Affiliations:** 1 Electronic Systems Group (GSE-UIB), University of the Balearic Islands, 07122 Palma, Spain; rafel.perello@uib.es (R.P.-R.); sebastia.bota@uib.es (S.B.); jaume.segura@uib.es (J.S.); 2 Health Research Institute of the Balearic Islands, IdISBa, 07010 Palma, Spain

**Keywords:** transimpedance amplifier, RF MEMS, oscillator, CMOS-MEMS

## Abstract

CMOS-MEMS resonators have become a promising solution thanks to their miniaturization and on-chip integration capabilities. However, using a CMOS technology to fabricate microelectromechanical system (MEMS) devices limits the electromechanical performance otherwise achieved by specific technologies, requiring a challenging readout circuitry. This paper presents a transimpedance amplifier (TIA) fabricated using a commercial 0.35-µm CMOS technology specifically oriented to drive and sense monolithically integrated CMOS-MEMS resonators up to 50 MHz with a tunable transimpedance gain ranging from 112 dB to 121 dB. The output voltage noise is as low as 225 nV/Hz^1/2^—input-referred current noise of 192 fA/Hz^1/2^—at 10 MHz, and the power consumption is kept below 1-mW. In addition, the TIA amplifier exhibits an open-loop gain independent of the parasitic input capacitance—mostly associated with the MEMS layout—representing an advantage in MEMS testing compared to other alternatives such as Pierce oscillator schemes. The work presented includes the characterization of three types of MEMS resonators that have been fabricated and experimentally characterized both in open-loop and self-sustained configurations using the integrated TIA amplifier. The experimental characterization includes an accurate extraction of the electromechanical parameters for the three fabricated structures that enables an accurate MEMS-CMOS circuitry co-design.

## 1. Introduction

Microelectromechanical systems (MEMS) resonators are becoming more and more used nowadays for RF and sensing applications. The use of these devices as the frequency-determining element in an oscillator circuit is a common demand in RF signal processing systems, leading to a reduction in area and power consumption [1]. Thanks to their small size and miniaturization capabilities, MEMS elements can prevent the use of discrete elements such as inductors and quartz crystals [2,3,4,5]. On the other hand, system-on-chip applications demand the integration of not only the MEMS signal conditioning circuitry, but also the electronics required to drive the mechanical system at resonance while continuously tracking its resonant frequency [6]. In this sense, the use of an oscillator circuit has additional advantages given its inherent capability of providing a pseudo-digital output signal rather than an analog one.

Various approaches can be found in the literature to implement resonators. It has been proven that resonant MEMS structures can be fabricated using standard CMOS processes combined with surface micromachining [1,6,7,8,9,10,11]. This approach enables the possibility of developing monolithically integrated systems that use electrostatic actuation and capacitive readout to drive and sense, respectively. the motion of a mechanical resonator, with benefits in terms of area reduction and noise performance.

However, when using CMOS technology, both the materials available to fabricate the MEMS structures as well as the technological design rules to define the MEMS sizes limit the electromechanical performance [10,12,13]. In the case of resonators, the scaling-down trend in the new generation of MEMS usually results in a reduction of the coupling capacitance between the moving structure and the sense electrode ($C_{sense}$), an increase in the resonant frequency, and a decrease in the quality factor. In addition, the maximum DC voltage ($V_{dc}$) applied between the moving and the fixed electrodes is limited by the pull-in voltage, or in any case, by the resonator linear operation range. As a consequence, the capacitive signal current, $I_{sig}$, injected in the sense electrode,

(1) $$I_{sig}=V_{dc}\frac{\partial C_{sense}}{\partial t},$$

is reduced to very low values. A transimpedance amplifier (TIA) with large gain and small input-referred noise, is typically used to convert $I_{sig}$ to a voltage feed at subsequent stages [14]. This voltage is often applied back to the resonator input with appropriate gain and phase to generate self-excited oscillations operating as parallel or series oscillators [6,8].

From these considerations, the motivation of this work is to develop a transimpedance amplifier suitable to drive monolithically integrated capacitive CMOS-MEMS resonators operating up to ~50 MHz and exhibiting large transimpedance gain (in the range of 10^6^ V·A^−1^) with a neglectable dependence on $C_{sense}$. The last provides a reliable tool for an accurate experimental characterization of MEMS resonators in the design stage, which might present an unknown or hard-to-predict equivalent parasitic capacitance value at the sense node, mostly associated with the MEMS resonator layout [15]. Thus, achieving a transimpedance gain independent of the fabricated structure is helpful for completing an accurate design and further parameter extraction during the redesign iterations accomplished to optimize the CMOS-MEMS required performance.

**Figure 1:**
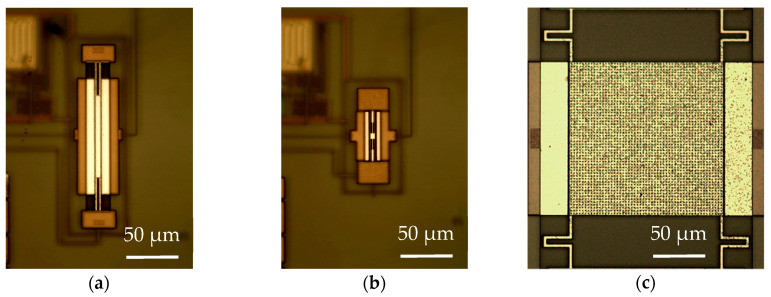
Optical image of the fabricated microelectromechanical system (MEMS) resonators: (**a**) PR1; (**b**) PR2; (**c**) PR3.

In this work, three MEMS resonators comprehending a broad range of dimensions (Figure 1) were designed, fabricated, and experimentally characterized by the addressed TIA amplifier obtaining its electromechanical parameters and comparing them with a theoretical model. Section 2 of this paper is devoted to the TIA amplifier, the theoretical model for the resonators is presented in Section 3, and the fabrication process is addressed in Section 4. Finally, Section 5 shows the experimental data collected and a summary of the work, and final conclusions and are given in Section 6.

## 2. Transimpedance Amplifier

A transresistance amplifier uses a resistor (passive or active) as the primary gain element to convert the MEMS capacitive current into a voltage. For high transresistance gains, the oscillator noise performance is usually quite poor and is dominated by the input resistor noise [10,16,17]. Thus, the two main types of CMOS oscillator topologies that have found practical applicability to MEMS resonators are the TIA-based oscillator [17] and the Pierce oscillator [18,19,20,21]. The TIA circuit converts the input current $I_{sig}$ into an output voltage $V_{o}$ that is used to self-excite the MEMS based oscillator circuit. The main challenge when designing a resonator-based oscillator is the quite large resonator-equivalent motional resistance $R_{M}$ (the circuit oscillates as long as the forward gain of the TIA overcomes the resonator series loss represented by $R_{M}$).

**Figure 2:**
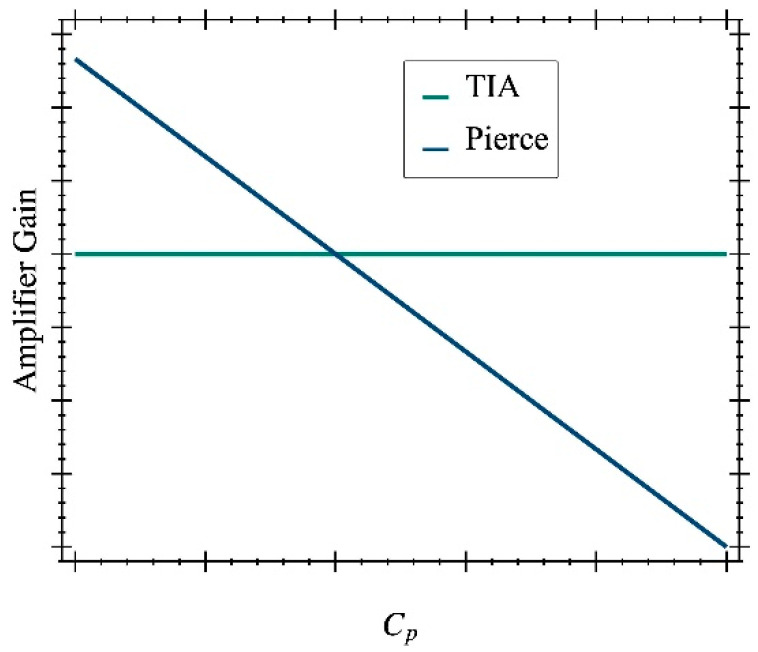
Open-loop gain from theoretical prediction for the two amplifier topologies as a function of the capacitance $C_{p}$.

The other alternative, the Pierce oscillator [18], uses a capacitive input to integrate the current from the resonator and convert it into a voltage at the sense node. The Pierce circuit topology is, in general, superior in terms of the oscillator noise figure since it exhibits an extremely high input-impedance [22,23]. Thus, the gain is provided by a noiseless capacitive input element rather than a lossy resistive element. One of the drawbacks of the Pierce oscillator topology is its inherent dependence of the open-loop gain with the parasitic capacitance $C_{p}$ at the sense node. This drawback does not represent a real limitation when the design is customized for a well-known resonator working under adequate operating conditions but lacks in versatility to deal with resonators with different characteristics since its contribution to $C_{p}$ is hard to accurately estimate from the resonator layout. Moreover, they are difficult to interface with high-impedance MEMS devices [17]. Figure 2 illustrates the expected dependence of the transimpedance gain vs. the parasitic capacitance $C_{p}$ for a both the TIA with capacitive feedback [17] like the one designed in this work and the behavior of the well-known dependence shown for a Pierce-based topology amplifier.

**Figure 3:**
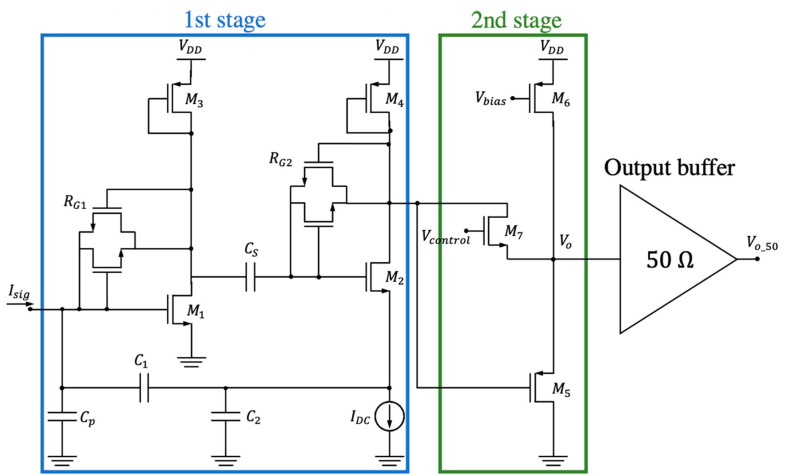
Schematic of the fabricated two-stage transimpedance amplifier (TIA) including an additional 50-Ω output buffer for testing purposes. The biasing resistors $R_{G1}$ and $R_{G2}$ have been implemented by two nMOS transistors in anti-parallel configuration.

In this work, we designed ad-hoc a commercial 0.35-µm 3.3 V CMOS technology TIA amplifier constituted by a first stage, based on the scheme from [16], and a second stage in which we added a control signal to tune the overall transimpedance gain as shown in Figure 3. The circuit behavior is closer to the ideal characteristic of Figure 2 in terms of sensitivity to the sense node parasitic capacitance.

In the first stage, $C_{p}$ corresponds to the resonator capacitance (including the routing components). $R_{G1}$ and $R_{G2}$ ensure that transistors $M_{1}$ and $M_{2}$ are saturated by appropriately biasing the transistor gate; given their high nominal values (they are in the range of GΩ), these devices have been implemented by two nMOS transistors, in anti-parallel configuration, working in their sub-threshold regions and consequently exhibiting an extremely high resistance [20]. In this design, we included a diode-connected pMOS transistor ($M_{4}$) with an equivalent small-signal resistance given by $R_{D}\approx 1/g_{m,M4}$. The transfer function can be written as

(2) $$\frac{V_{o}\left(s\right)}{I_{sig}\left(s\right)}=\frac{I_{out}\left(s\right)}{I_{sig}\left(s\right)}R_{D}\approx \left(1+\frac{C_{1}}{C_{2}}\right)R_{D},$$

to obtain a gain close to 2.5 × 10^6^ V·A^−1^ with $R_{D}$ = 36 kΩ, $C_{2}$ = 6 pF and $C_{1}$ = 90 fF.

The second stage is a pMOS-based source follower with shunt-shunt feedback implemented through a nMOS device working in the linear region. The gate voltage is externally controlled through $V_{control}$, which can take values between 0.5 and 2.5 V. This nMOS plays the role of variable resistance, providing tunable input resistance in terms of $V_{control}$. This variable input resistance affects the output resistance of the first stage and consequently its gain (Figure 3) [19].

**Figure 4:**
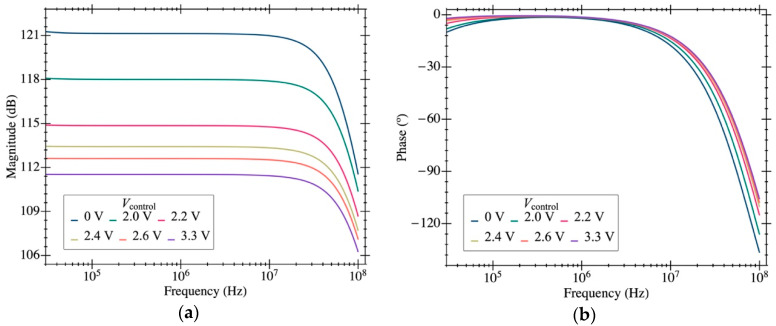
Bode plots of amplitude (**a**) and phase (**b**) of the proposed TIA at the $V_{o}$ node. Results have been obtained under nominal biasing conditions with $C_{p}$ = 20 fF.

**Figure 5:**
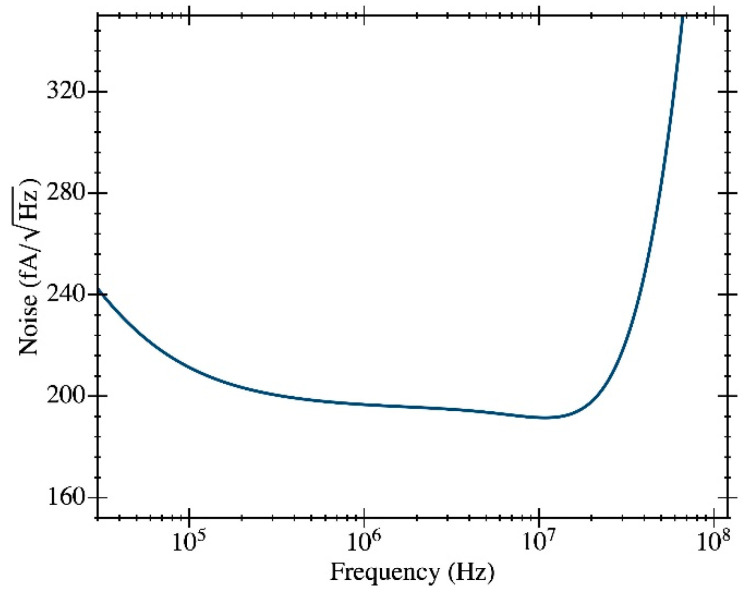
Simulation results for the input-referred current noise of the current TIA design.

Several simulation analyses were carried out to characterize the performance of the proposed circuit. Figure 4a shows the frequency response of the transimpedance gain (magnitude and phase). A maximum gain of 1.17·10^6^ V·A^−1^ (121 dB) was achieved over a bandwidth of 50 MHz. We notice that this value is lower than the 2.5·10^6^ V·A^−1^ obtained from Equation (2) given the moderate voltage gain for the first stage used in our design. Figure 4a also reports the dependence of the gain with $V_{control}$ (a very low impact has been observed in the phase plot in Figure 4b). The gain can be adjusted between 112 and 121 dB, varying $V_{control}$ between 0 and 3.3 V. Figure 5 shows the input-referred current noise, having a mean value of 192 fA·Hz^−1/2^ at frequencies around 10 MHz (equivalent to 225 nV·Hz^−1/2^ at the output). A low power consumption value of 930 µW for a 3.3 V voltage supply was obtained.

Table 1 compares this TIA design to prior works [14,17,24,25,26,27]. As stated earlier, in addition to the gain insensitivity to $C_{p}$, a significant advantage of the proposed solution features a tunable-gain option. Finally, a Figure of Merit (FoM) is devised to compare the circuit design effort in series-resonant oscillators with various oscillating frequencies and resonator characteristics [14]:

(3) $$FoM=\frac{k_{B}T}{V_{on}^{2}}\frac{BW^{2}R_{M}^{2}}{P},$$

where $k_{B}$ is the Boltzmann constant, T is the absolute temperature, $V_{on}$ is the output noise, BW is the TIA bandwidth, $R_{M}$ is the TIA transimpedance gain, and P is the power consumption. The amplifier developed in this work exhibits an FoM value of 3.04·10^23^, which is absolutely competitive with the reported state of the art as shown in Table 1. For comparison with the Pierce oscillator topology, an FoM value of 1.06·10^27^ was achieved for a highly compact Pierce-based amplifier integrated together with a seesaw resonator operating at frequencies below the 1-MHz range [21]. Despite the fact that it provides better performance compared to the present work, this would be rapidly degraded for higher operating frequencies and particularly for larger resonator parasitic capacitances. Similarly, in [23], a Pierce oscillator has been also demonstrated to achieve GSM noise requirements specifically oriented to phase noise optimization with a solution fabricated using the very same technological approach as this current work.

## 3. Oscillator Electromechanical Model

**Figure 6:**
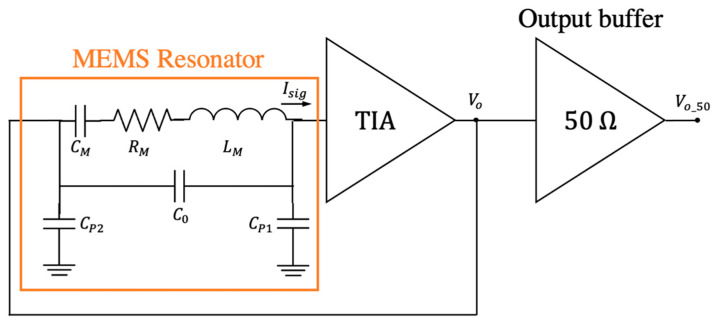
Oscillator schematic: MEMS resonator (represented by its lumped electrical equivalent model) and the transimpedance amplifier (TIA).

The oscillator is constituted by the MEMS resonator, represented by its lumped equivalent RLC circuit and the feedthrough capacitance between the actuation and readout drivers [28], connected in series to the TIA as shown in Figure 6. In addition, an output buffer for testing purposes was also included in the integrated circuit scheme.

The equivalent admittance ($Y_{eq}$) in terms of the lumped elements is:

(4) $$Y_{eq}={\left(j\omega L_{M}+\frac{1}{j\omega C_{M}}+R_{M}\right)}^{-1}+j\omega C_{0},$$

where the feedthrough capacitance ($C_{0}$) has also been included. The open-loop configuration amplifier output voltage is obtained from the equivalent admittance and the transconductance gain (G), which is accurately determined from the TIA amplifier post-layout simulations:

(5) $$V_{o}=GY_{eq}.$$

The main goal of this work is to determine these parameters for any specific resonator from an open-loop response by model fitting considering that the amplifier gain roughly depends on the resonator driver parasitic capacitance. From a practical point of view, it is advisable to use Q and $\omega_{0}$ as fitting parameters, together with $R_{M}$, instead of $C_{M}$ and $L_{M}$, which can be computed afterward. Therefore, using these definitions from the RLC branch,

(6) $$Q=\frac{1}{\omega_{0}R_{M}C_{M}},$$

(7) $$\omega_{0}=\frac{1}{\sqrt{L_{M}C_{M}}},$$

the output voltage is fitted from the open-loop response in terms of $\omega_{0}$, Q, $C_{0}$, and $R_{M}$:

(8) $$V_{o}=G\left(\frac{j\frac{\omega }{\omega_{0}R_{M}Q}}{1-{\left(\frac{\omega }{\omega_{0}}\right)}^{2}+j\frac{\omega }{\omega_{0}Q}}+j\omega C_{0}\right)$$

Therefore, the resonator is fully characterized by first fitting the parameters in Equation (8) and subsequently computing the remaining parameters ($C_{M}$ and $L_{M}$). The motional resistance represents the crucial parameter as it determines the amplifier gain constraints for a self-sustained oscillator.

## 4. MEMS Fabrication and Design

**Figure 7:**
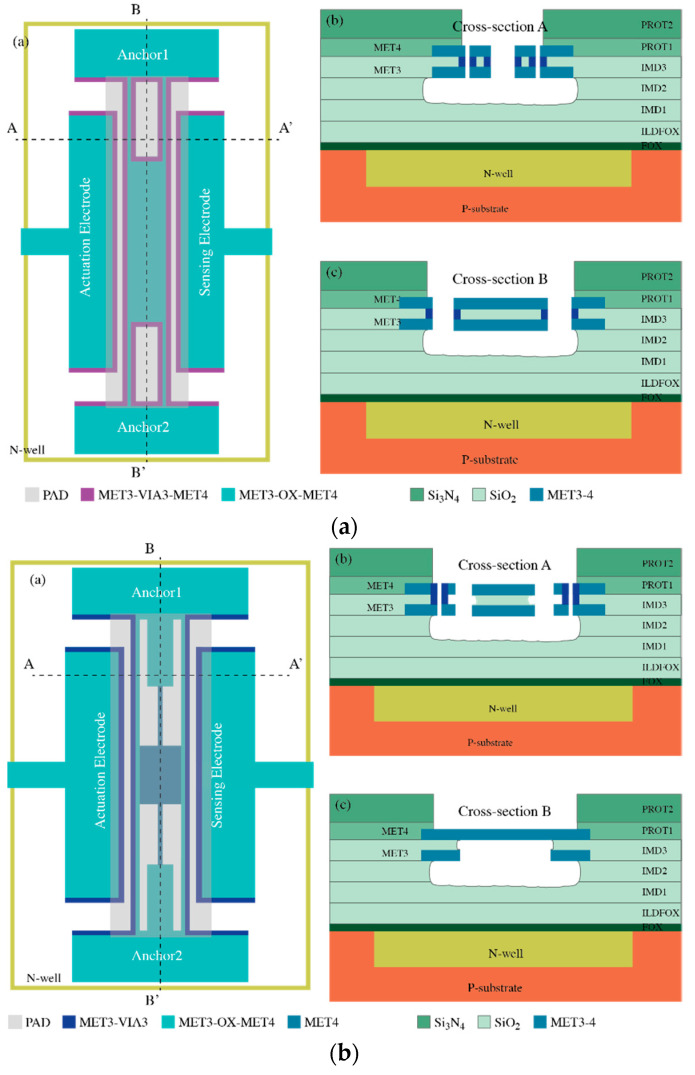

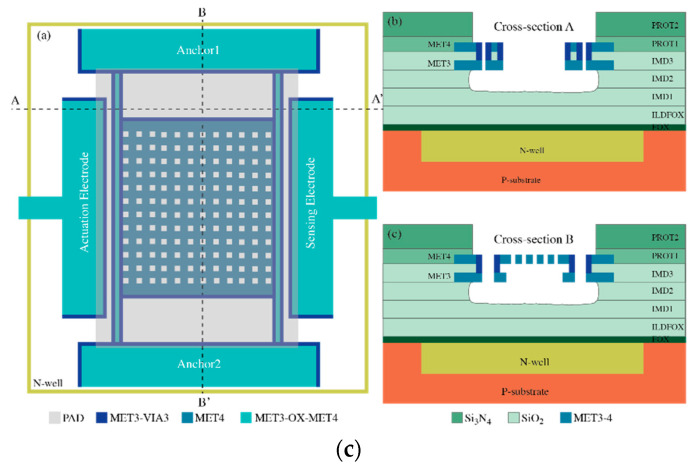
Schematic design of the fabricated MEMS resonators including the different layers that have been used: (**a**) PR1, (**b**) PR2 and (**c**) PR3.

The mechanical resonators measured in this work were fabricated using a commercial CMOS 0.35-μm technology with an additional post-CMOS step consisting of a mask-less wet-etching performed at our laboratory. This additional step is aimed to release the resonators by removing the sacrificial oxide layer underneath, obtaining a fully integrated CMOS-MEMS solution [29]. The CMOS process used provides two polysilicon layers and a stack of four metal layers (TiN-Al-TiN) interconnected by Tungsten (W) vias. This fabrication approach based on a CMOS commercial technology provides a wide range of benefits as detailed in [9,10]. However, only the materials available in the manufacturing process can be considered as structural layers, and the manufacturer design rules must also be conformed to [10,12,30]. Thus, the resonator performance is constrained in some issues as a poor-quality factor (Q) compared to specific MEMS fabrication processes, reduced capacitive coupling between the resonator and the readout driver due to fixed layer thickness and minimum distance, and a relatively high device temperature sensitivity. Three design options, shown in Figure 7, were considered in this work, called Plate Resonator 1 (PR1), Plate Resonator 2 (PR2), and Plate Resonator 3 (PR3). All of them consist of an anchored plate resonator with a two-driver configuration with electrostatic actuation and capacitive readout to make it feasible to combine the MEMS with the readout circuitry in the same IC for a monolithic integration [31]. The large motional resistances exhibited by the resonators (as a consequence of the aforementioned relatively small capacitive coupling) is compensated by the high-gain, low-noise integrated TIA and a significant reduction of the parasitic contributions.

The MEMS resonators were designed by means of a two-layer strategy, similar to that in [32], so that the coupling capacitance became larger compared to single-layer geometries, i.e., decreased the motional resistance. The coupling can be improved by either reducing the resonator-driver gap or by increasing the composite material thickness. Therefore, the first two structures presented here exploit each one of these alternatives for design optimization. Additionally, a composite material in the form of metal–oxide–metal reduces its overall temperature sensitivity because the oxide relative stress when increasing the operating temperature changes opposite to that of the metal layer [33]. This fact is also a key aspect for MEMS resonators design when considering the final oscillator stability as an important figure of merit directly related to the sensor limit of detection. In this sense, the PR1 structure benefits from MET3-OX-MET4 and MET3-VIA-MET4 layers (see Figure 7a), while the PR2 was designed to have a MET-VIA stack as in Figure 7b. On behalf of PR3, it inherits most of the design strategy developed for PR2 shown in Figure 7c. It scales up its platform dimensions so as to provide a solution suitable for inkjet deposition; one of the possible applications for this resonator is to operate as a gravimetric gas sensor thanks to its outstanding distributed mass sensitivity [31] together with a proper functionalization process with a specific layer to capture the target molecules. A detailed discussion of each structure is presented next.

### 4.1. Plate Resonator 1

This design, shown in Figure 7a, consists of a four-anchored plate resonator composed of two materials: (i) the resonator main body is designed to be manufactured with a MET3-OX-MET4 (MOM) compound so that the overall effective mass growth is minimum compared to using the VIA layer required in the anchors and drivers for coupling requirements; (ii) the anchors combine the previous stack with a MET-VIA-MET (MVM) composite to get an increase in the readout coupling thanks to the larger thickness when including MET4. However, the minimum distance between two MET4-adjacent layers is 600 nm, worsening the overall coupling when compared to a gap of 500 nm available by design rules when using VIA or lower metal layers. To investigate this option, we created the second structure (Figure 7b), where the MV composite is not used in the whole beam to avoid an excessive increase of the whole resonator mass density [32].

### 4.2. Plate Resonator 2

The device shown in Figure 7b is a six-anchored plate resonator with the four outer anchors conceived to provide large coupling and vibration stability, while the two inner ones increase the overall stiffness to achieve higher oscillation frequency. Similarly to the previous design, it also combines various material aggregates: (i) the main body and the inner anchors were made of a single MET4 (M) layer as it does not play any role in the coupling capacitance—not requiring an increased thickness—while the overall mass is reduced, thus decreasing the motional resistance; (ii) the outer anchors merge the MOM composite with a MET3-VIA composite at the driver side to reduce the resonator-driver gap since MET3 and VIA layers can have a separation of only 500 nm. A reduction in the gap has a higher impact on the coupling than the overall thickness does. In any case, the coupling thickness of this structure will be smaller than the one of the previously described structure. Notice that, depending on the metal and via combinations, the thickness of each layer may vary [34].

The structure depicted in Figure 7c takes the design strategy from PR2 and implements some improvements in the pursuit of a large enough platform to enable inkjet deposition featuring droplets with diameter in the order of 70 µm. It combines the same material mixtures with a single metal layer approach: (i) the main body material is the MET4 layer to reduce the overall mass and, in consequence, the motional resistance, as well as to allow for regularly spaced holes for release purposes; (ii) the beams combine the MOM composite in the center for temperature insensitivity with the MV in both sides to increase the stiffness and improve the capacitive coupling; and (iii) the perimeter of the structure also features the MV composite to provide an increased stiffness to the overall platform and avoid vertical bending under self-loading effects. According to manufacturer fabrication parameters, the readout electrode parasitic capacitance featured for this structure is in the order of 80 fF; 8× the value found for a minimum-size MEMS resonator fabricated with the same 0.35-µm technology [15]. Thus, one would expect for the Pierce topology alternative to have its open-loop gain reduced, similar to the possibility of failing to compensate for the motional resistance losses and, consequently, not being able to operate as a self-sustained oscillator. In such a case, it is the TIA alternative that provides a huge advantage, having an open-loop gain that does not diminish with increased input capacitance. Therefore, in this work, we will also provide electrical characterization for this structure obtaining a parameter extraction with an accuracy that, otherwise, would have been not feasible with a Pierce scheme-based amplifier.

## 5. Electrical Characterization

### 5.1. Transimpedance Amplifier

**Figure 8:**
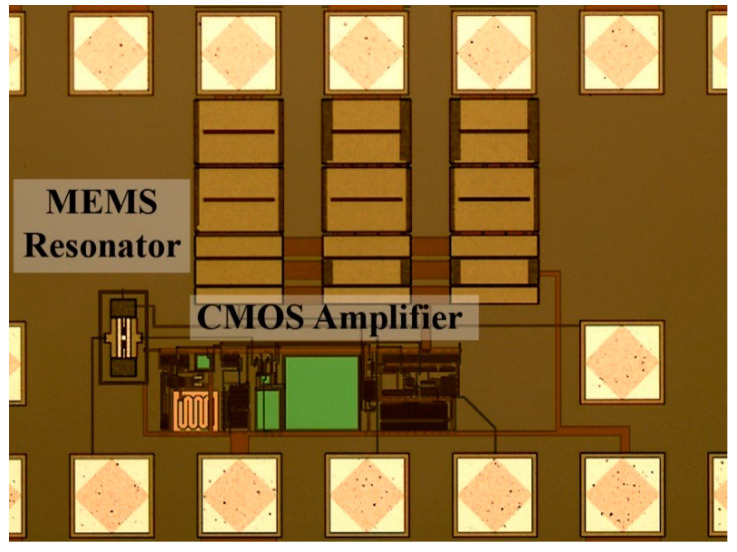
Optical image of the CMOS-MEMS oscillator IC fabricated in a CMOS 0.35-μm commercial technology.

**Figure 9:**
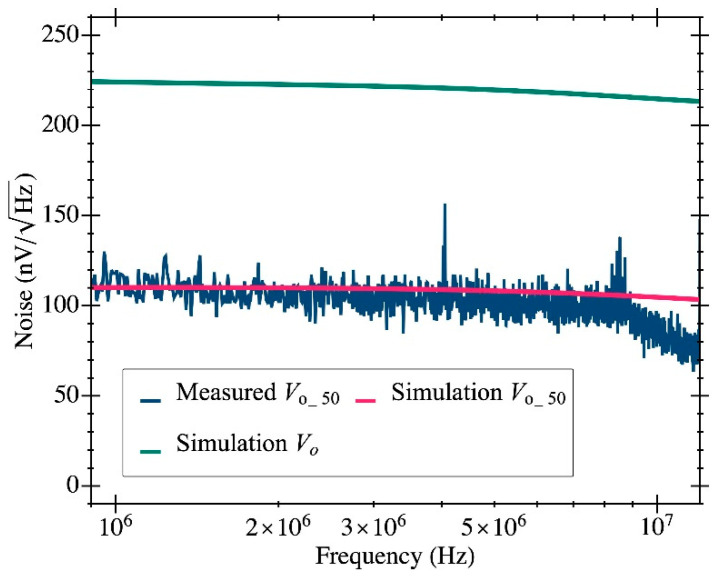
The measured output voltage noise (blue line) at $V_{o\_50}$ is compared to the simulation results at the same node (magenta line) to show that both values are in good agreement. Additionally, the simulated voltage noise at $V_{o}$ is depicted with a solid green line to show how the output buffer attenuates the voltage signal.

Figure 8 shows one the CMOS-MEMS devices fabricated in the IC using a CMOS 0.35-μm commercial technology. First, the CMOS TIA circuit was characterized in terms of noise performance. A voltage noise at the $V_{o\_50}$ node of 110 nV/Hz^1/2^ at 10 MHz was measured, providing a value that perfectly matches the simulation outcome (see Figure 9). For clarification, Figure 9 also depicts the simulated voltage noise at the $V_{o}$ node (the output of the TIA 2nd stage), which is 225 nV/Hz^1/2^ at 10 MHz (see Table 1). This node is the one used to drive the resonator excitation and closes the oscillator loop, but it cannot be experimentally tested since it was not designed to interface with the 50-Ω input laboratory benchtop equipment [20]. Notice that the noise at this node is higher than the one obtained at the tested node $V_{o\_50}$ because of the attenuation introduced by the output buffer.

### 5.2. Plate Resonator 1 and Plate Resonator 2

The CMOS-MEMS resonators were characterized in both open- and closed-loop configurations thanks to the capacitive readout scheme and the integrated amplifier. Figure 9 shows the measured electromechanical transmission coefficient (obtained with a *Keysight ENA E5061B* network analyzer) at low excitation power (−35 dBm) to keep the resonators’ operation in the linear regime both for vacuum (below 10^−3^ mbar) and ambient pressure. Additionally, a sweep of the resonator DC biasing voltage from 10 V to 40 V was performed to obtain the motional resistance at various values and experimentally observe the relationship in which the resistance is inversely proportional to the squared DC voltage through the electromechanical coupling.

**Figure 10:**
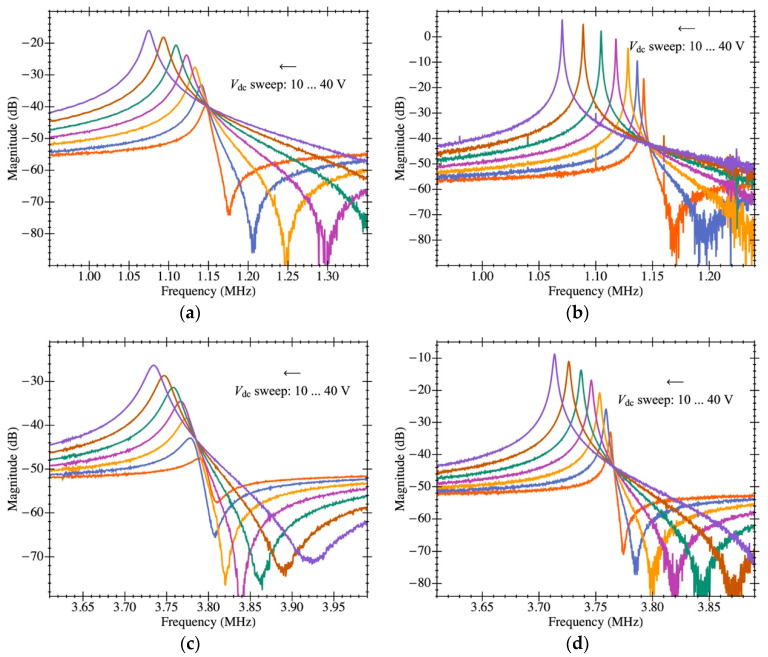
Electrical characterization of the MEMS resonator with on-chip readout circuit in open-loop configuration. Experimental magnitude of frequency response for various MEMS bias voltages (*V_dc_*): (**a**) plate resonator PR1 in ambient pressure; (**b**) plate resonator PR1 in vacuum pressure; (**c**) plate resonator PR2 in ambient pressure; (**d**) plate resonator PR2 in vacuum pressure.

**Figure 11:**
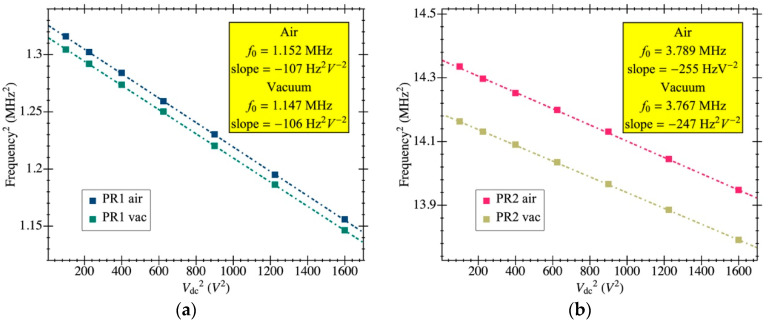
Resonance frequency dependence with the resonator bias voltage obtained from experimental open-loop response data fitting. The linear fit for the frequency values is also included (dashed lines) to obtain the natural frequency at 0 bias voltage for each case: (**a**) PR1; (**b**) PR2.

**Figure 12:**
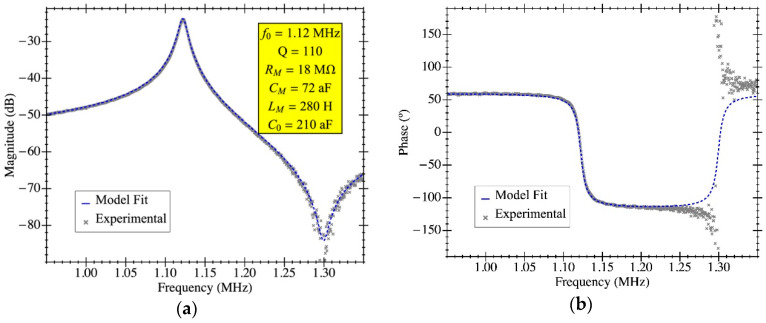
Electrical characterization of the MEMS resonator with on-chip readout circuit in open-loop configuration for RLC parameter extraction by means of model fitting. The experimental data corresponds to PR1 using a bias voltage of 25 V in air conditions: (**a**) magnitude and (**b**) phase.

**Figure 13:**
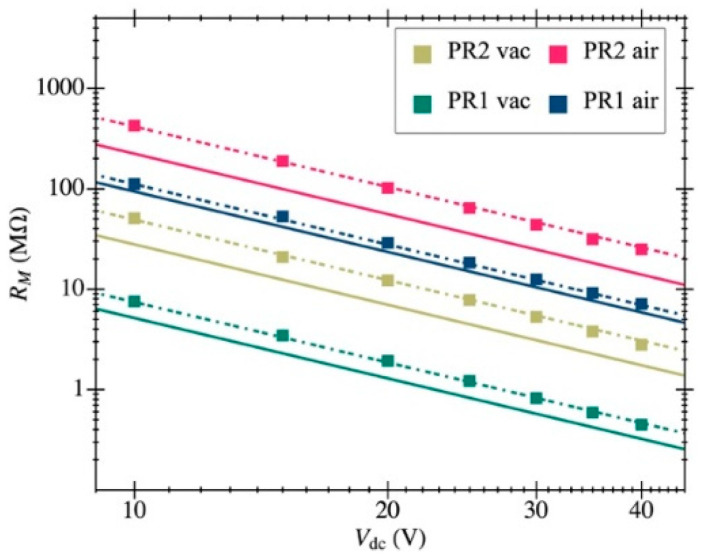
Motional resistance computed from experimental open-loop data by means of the proposed model fit: the value is inversely proportional to the square of the resonator biasing voltage. The obtained fit (dashed lines) matches the theoretical prediction (solid lines) for both structures in air and vacuum conditions.

The experimental response shown in Figure 10 was fitted to the model in Section 3 to obtain the aforementioned parameters. The resulting resonance frequency is plotted in Figure 11 as a function of the biasing voltage and fitted as $f^{2}=a-bV_{dc}^{2}$, from which the natural frequency was computed (resonant frequency at 0 V biasing). Figure 12 illustrates an example of such fitting with the extracted values demonstrating high model accuracy, as it perfectly matches the measured transmission coefficient. Remarkably, this degree of precision in the parameter extraction procedure is only achievable thanks to having an amplifier open-loop gain that does not depend on the input capacitance and whose specific value was obtained from post-layout simulations. The parameters obtained for both resonators are listed in Table 2 for a biasing voltage of 25 V. We also obtained the motional resistance for all biasing voltages (plotted in Figure 13); the theoretical prediction is also included in solid lines as well as the linear fitting in logarithmic scale in dashed lines. Results show that the theoretical motional resistance prediction matches the experimental data with an error below 20% in the worst case; such a reliable calculation is also a direct consequence of the amplifier gain being accurately characterized. In any case, the remaining error could be attributed to the metal layers’ degradation caused by the etching step that is intended to remove only the sacrificial oxide but in practice has an impact on the metal layers as well [35]. Such a metal degradation can reduce the overall resonator-driving coupling capacitance, resulting in experimental motional resistances larger than expected. Furthermore, the fabrication process itself, together with the post-CMOS etching step, has been shown to produce a 10% error in the measured resonant frequency, which goes in line with the error also observed for the motional resistance.

**Figure 14:**
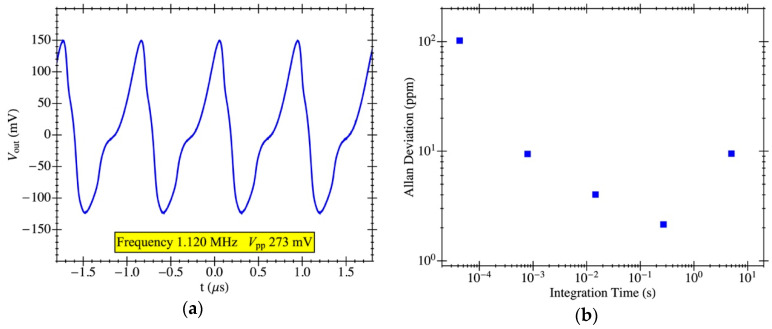
MEMS resonator electrical characterization with on-chip CMOS amplifier in a closed-loop configuration: (**a**) oscillator output voltage in the time domain for PR1 with a biasing voltage of 25V; (**b**) measured Allan deviation as a function of the integration time in ambient temperature and pressure.

The PR1 structure was also measured in closed-loop configuration to operate as an oscillator in self-excited mode in vacuum conditions obtaining the output voltage waveform shown in Figure 14a. The self-sustained oscillation started at a DC biasing voltage of 23 V, and at *V_dc_* = 25 V, the oscillator exhibited a frequency of 1.120 MHz and a peak-to-peak voltage of 273 mV. The measured Allan deviation (obtained with the frequency counter Pendulum CNT-90) is also provided in Figure 14b for integration times ranging from 5·10^−5^ s to 5 s, reaching a minimum value of 2 ppm for an integration time of 300 ms. The experimental value for the oscillator stability is close to the one obtained in previous works using a Pierce topology for the oscillator circuit [36].

### 5.3. Plate Resonator 3

**Figure 15:**
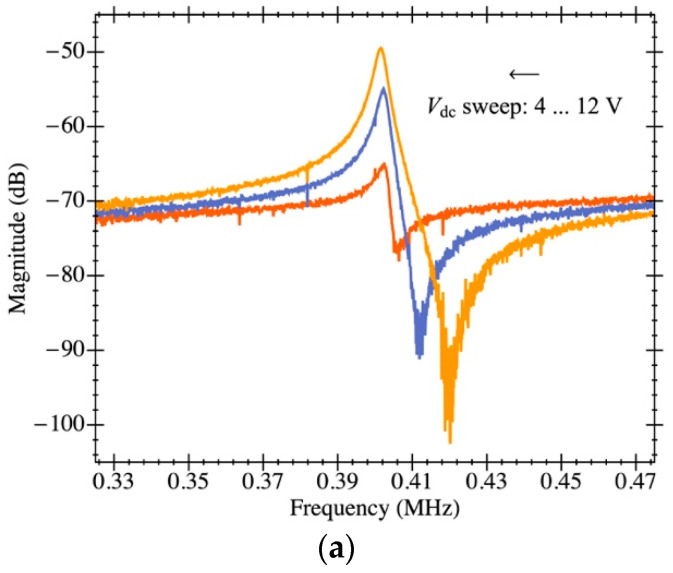

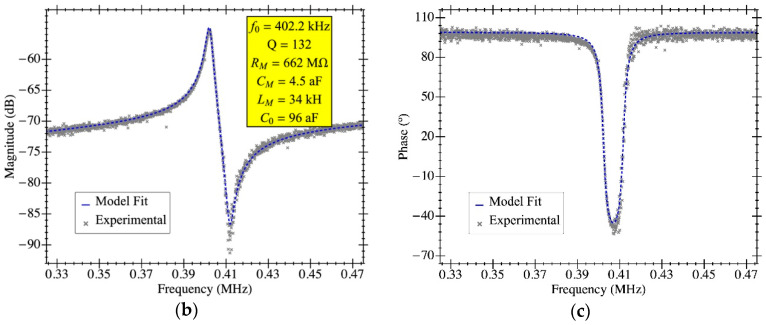
Electrical characterization of the MEMS resonator with on-chip readout circuit in open-loop configuration. (**a**) Experimental magnitude of frequency response for various MEMS bias voltages (*V_dc_*) for the plate resonator PR3 in ambient pressure. RLC parameter extraction by means of model fitting; the experimental data corresponds to a bias voltage of 8 V in air conditions: (**b**) magnitude and (**c**) phase.

In this section, the extraction of electromechanical parameters for PR3 are also obtained using the capabilities of the TIA amplifier addressed in this work. The plate resonator has a 150 µm × 150 µm platform that features a parasitic capacitance due to the readout driver on the order of 80 fF estimated from manufacturer specifications. The open-loop response in air conditions is shown in Figure 15a for a biasing voltage that ranges from 4 V to 12 V. Additionally, the Bode plot corresponding to 8 V biasing is depicted in Figure 15b,c and fitted to the theoretical model presented. It is clearly shown that both the experimental curve and theoretical model are in good agreement thanks to having the open-loop gain of the on-chip amplifier perfectly characterized and not depending on the aforementioned parasitic capacitance. Interestingly, the feedthrough capacitance obtained for PR3 is 2× smaller than the one featured by PR1 in spite of having a much larger platform size. Actually, this is directly related to the fact that, with a larger platform, the readout and driving electrodes are further dissociated and, in consequence, the parasitic capacitance between them is smaller. Additionally, the obtained motional resistance for such a structure is well above the one presented for PR1—662 MΩ over 18 MΩ—as a consequence of the highly increased mass.

## 6. Conclusions

The design of capacitive readout circuits specifically oriented to CMOS-MEMS resonators that operate in the MHz-range is a demanding task if state-of-the-art outcomes are to be achieved due to moderate capacitive coupling and rather poor-quality factor of fabrication materials. In this work, we have provided experimental proof of a design that not only enables self-sustained oscillation but also provides a great characterization capability together with tunable gain so as to keep resonators operating in their linear regime that suits a wide range of resonator characteristics.

We developed a versatile transimpedance amplifier (TIA) designed in a commercial 0.35-μm 3.3 V CMOS technology specifically oriented to monolithic CMOS-MEMS resonators. The proposed design is clearly competitive with the state of the art, achieving low power, low noise, and a high gain that enables closed-loop oscillation of MEMS resonators that have been experimentally tested. Moreover, it offers tunable gain between 112 and 121 dB with a dramatically reduced dependence on the input parasitic capacitance compared to other common alternatives such as the Pierce topology commonly used in previous works [37].

The TIA was applied to accurately characterize three MEMS resonators monolithically fabricated into the same CMOS die. The extraction of their electromechanical parameters using the theoretical model presented offers an extremely precise fitting, thus being able to obtain a correct value for the motional resistance thanks to having a parasitic capacitance independent gain. This value represents a key parameter in MEMS resonator and sustaining amplifier co-design, being of great importance in the design stage for future fabrication iterations and system improvement.

## Figures and Tables

**Table 1:** Performance comparison with state-of-the-art reported works based on a TIA topology.

TIA	[24]	[14]	[25]	[17]	[26]	[27]	This Work
Bandwidth	280 MHz	20 MHz	60 MHz	1.8 MHz	1.2 MHz	90 MHz	50 MHz
Gain	25.0 kΩ–89.0 kΩ	12.0 kΩ–69.0 kΩ	316 kΩ	56.0 MΩ	7.94 MΩ	12.5 kΩ–794 kΩ	330 kΩ–1.17 MΩ
Output Voltage noise	-	-	790 µV/Hz^1/2^	3.64 µV/Hz^1/2^	199 nV/Hz^1/2^	3.18 µV/Hz^1/2^	225 nV/Hz^1/2^
Input-Referred Current Noise	-	-					192 fA/Hz^1/2^
CMOS Technology	0.18 µm	0.35 µm	0.18 µm	0.18 µm	0.35 µm	65 nm	0.35 µm
FoM	-	-	4.04·10^14^	7.28·10^21^	6.65·10^22^	1.66·10^20^	3.04·10^23^
Power Consumption	1.57 mW	6.90 mW	5.90 mW	436 µW	150 µW	900 µW	930 µW

**Table 2:** Extracted parameters from experimental open-loop response by model fitting for both resonators in air and vacuum conditions.

Resonator	f_0_ (MHz)	Q	R_M_ (MΩ)	C_M_ (aF)	L_M_ (H)	C_0_ (aF)
PR1 (air)	1.152	110	18 ^†^	72 ^†^	280 ^†^	210
PR1 (vac)	1.147	2100	1.2 ^†^	56 ^†^	360 ^†^	210
PR2 (air)	3.789	210	63 ^†^	3.4 ^†^	530 ^†^	890
PR2 (vac)	3.767	1600	7.7 ^†^	3.3 ^†^	520 ^†^	890
PR3 (air)	0.402	132	662 ^§^	4.5 ^§^	34,000 ^§^	96

^†^ These values refer to a biasing voltage of 25 V. ^§^ These values refer to a biasing voltage of 8 V.
